# Effect of wood kraft pulp feed on digestibility, ruminal characteristics, and milk production performance in lactating dairy cows

**DOI:** 10.1111/asj.13131

**Published:** 2018-12-16

**Authors:** Keiko Nishimura, Kazuhiro Kurosu, Fuminori Terada, Hitoshi Mizuguchi, Shigeru Sato, Shiro Kushibiki

**Affiliations:** ^1^ Miyazaki Livestock Research Institute Miyazaki Japan; ^2^ Nippon Paper Industries Co. Ltd. Tokyo Japan; ^3^ Faculty of Agriculture Tohoku University Sendai Japan; ^4^ DKK‐TOA Yamagata Corporation Yamagata Japan; ^5^ Faculty of Agriculture Iwate University Morioka Japan; ^6^ Institute of Livestock and Grassland Science NARO Tsukuba Japan

**Keywords:** kraft pulp, LPS, milk production, ruminal pH, SARA

## Abstract

The effect of wood kraft pulp (KP) feed on dietary digestibility, ruminal fluid pH, rumen fermentation characteristics, and milk production performance in lactating dairy cows was examined. Four lactating dairy cows were used for the feeding experiment by the cross‐over design. The control group and KP group were set up as treatments. The control group was fed total mixed ration (TMR) (40% roughage and 60% concentrate) and the KP group was fed TMR containing 12% KP that replaced half of the rolled corn in the control diet. The dry matter intake, digestibility of the feed components, and milk yield were not significantly different between control group and KP group. The number of times that the ruminal fluid pH was below 6.1 tended to decrease in the KP group compared to the control group (*p *<* *0.10). The acetic acid ratio in the ruminal fluid of the KP group increased compared to the control group (*p *<* *0.05) and the propionic acid ratio in the ruminal fluid of the KP group decreased compared to the control group (*p *<* *0.05). The acetate:propionate acid ratio was increased in the KP group compared with the control group (*p *<* *0.05). Lipopolysaccharide levels in the ruminal fluid of the KP group tended to decrease compared to the control group (*p *<* *0.10). Based on these results, it was indicated that the use of KP feed for lactating dairy cows induced the same rumen fermentation characteristics as those in cows given a large amount of roughage without depressing milk productivity. Therefore, KP could be a valuable feed resource substitute for grains, which would also reduce the risk for subacute rumen acidosis.

## 1 INTRODUCTION

There has recently been a remarkable improvement in the lactation performance of dairy cows, and in 2014, the average annual milk production per lactating dairy cow reached 9,382 kg in Japan (LIAJ [Livestock Improvement Association of Japan], 2014). The improvement in lactation performance was achieved not only by genetic advancements, but also by nutrition management that relies on a large amount of concentrate supplementation. The annual average amount of concentrated feed that is supplied to lactating dairy cows across prefectures other than Hokkaido is 3,806 kg per cow, which represents an increase of 184.1% compared to feed consumption in 1975 (LIAJ, 2014). However, as the main ingredients of such feed are based on imported grains, the price of concentrated feed tends to fluctuate in accordance with international grain markets, and the price has recently remained high (MAFF (Ministry of Agriculture, Forestry and Fisheries Japan), 2016). To stabilize the feed cost, it is necessary to secure feed resources that can be supplied domestically.

One potential solution is to consider wood‐based resources for use in feed. Nearly 67% of the land in Japan is forested (MAFF, 2014). There have been several studies on the utilization of wood‐based feed for cows (Kajikawa et al., 1987; Nakagawa, Fukuyama, Kawamura, Niimi, & Kawamura, 2009; Nakagawa, Fukuyama, Kobayashi, Mini, & Kawamura, 2010; Tsuneishi, Takimoto, Nishimura, & Watanabe, 1988), and although wood‐based materials contain lignin which interferes with digestion, digestibility increases with a steaming treatment (Terada et al., 1988) and the possibility of using the steamed wood as a roughage replacement has been highlighted. In contrast, lignin is attached to the fiber of wood, and lignin must be removed using chemical methods for making pulp from wood chips. The kraft process, which uses sodium hydroxide and sodium sulfide to make pulp wood, is the dominant pulping process in the pulp and paper industry. Therefore, wood kraft pulp (KP) is a cellulosic pulp in which the lignin has been removed from wood chips using kraft process treatment. Because the total digestible nutrient (TDN) content of KP is equivalent to rolled corn (Hada, Yashiro, Machida, & Kajikawa, 2016; MAFF, 2012), there is the possibility that KP can be used as a replacement of concentrated feed. Therefore, in this study, the effect of feeding KP on lactating dairy cows in terms of its digestibility, rumen fluid pH, rumen fermentation characteristics, and milk production was evaluated.

## 2 MATERIALS AND METHODS

The research protocol regarding animal use followed the guidelines recommended in the Standards relating to the Care and Keeping of Industrial Animals (Notice the Prime Minister's Office No. 22 of 1987).

### 2.1 Animal and experimental design

Four lactating Holstein cows 614 ± 57 kg body weight; 195 ± 40 day in milk; (*M* ± *SD*) were used in this experiment. Cows were assigned to experimental treatments as a cross‐over design. Treatments were a control group or a KP group. The control group was fed total mixed ration (TMR) without KP (control diet) and the KP group was fed TMR that contained KP (KP diet). In the KP diet, KP replaced 50% of the rolled corn that was contained in the control TMR, resulting in a mix ratio dry matter (DM) of 12% (Table 1). KP was supplied by Nippon Paper Industries Co., Ltd., in fluffy form. The diets were formulated to meet or exceed the energy and protein requirements of Holstein cows (Agriculture, Forestry and Fisheries Research Council Secretariat, 2006). Cows were fed one‐half of the daily allowed feed at 11:00 a.m. and the other half at 17:00 p.m. The allowance TMR was 110% of the previous day's intake. Cows had free access to fresh water and mineral blocks during the trial. The cows were housed in a tie stall barn during the experimental period. Each experimental period consisted of 11 day of adaptation to the feed and 3 day of experimental measurements. Individual diets samples were collected before feeding everyday over 3 day of the experimental period for analysis of chemical composition. Total orts were collected everyday over 3 day of the experimental period. Grab samples of feces were taken twice daily (at 07:00 a.m. and 16:00 p.m.) during the experimental period. The apparent digestibility was measured using acid detergent lignin (ADL) as external marker according to the procedure provided by GAFSA (Japan Grassland Agriculture and Forage Seed Association) (2009).

**Table 1:** Ingredients composition in the diets of control group and KP group

	Control group	KP group
Ingredients (% DM)		
Corn silage	10.0	10.0
Itarian rygrass silage	5.0	5.0
Alfalfa hay	15.0	15.0
Oat hay	10.0	10.0
KP	—	12.0
Steam rolled corn	24.0	12.0
Steam rolled barley	21.0	20.0
Soybean meal	8.5	11.5
Wheat bran	5.6	3.6
Calcium carbonate	0.7	0.7
Vitamin ADE	0.2	0.2

*Notes*.

KP: kraft pulp; DM: dry matter.

### 2.2 Sample analyses

The offered feed, orts, and fecal samples were dried (at 60℃, for 24 hr), weighed, and ground through a 1‐mm screen using a Willy mill (Retsch SM2000; F. Kurt RetschGmbH&Co. KG, Germany). Samples were analyzed for DM, crude protein (CP), ether extracts (EE) and crude ash (GAFSA, 2009), and neutral detergent fiber was determined using heat stable amylase and expressed as exclusive of residual ash (aNDF; Van Soest, Robertson, & Lewis, 1991). The content of ADL was determined (GAFSA, 2009). The feed starch concentration was determined using a colorimetric procedure (Total starch assay kit, Megazyme International Ireland Ltd., Wicklow, Ireland; McCleary, Solah, & Gibson, 1994). CP in fresh fecal samples was also analyzed (GAFSA, 2009).

### 2.3 Rumen pH measurement

The ruminal fluid pH was measured continuously (10‐min intervals) throughout the 10 day of the experimental period by the radio‐transmission pH‐measurement system (YCOW; DKK‐Toa Yamagata), which consists of a wireless pH sensor that was developed for the purpose of various similar studies (Kimura et al., 2012; Sato, Ikeda, et al., 2012; Sato, Kimura, et al., 2012). A pH reading was taken every 10 min, with averages of every 1 hr period calculated. The sensors were administered orally to each of the four cows. Because this sensor was held in the bottom of the reticulum, the measured value indicated the pH of the reticulum fluid. The pH of the reticulum was higher than that of the rumen by an average 0.3–0.5 (Sato, 2016). Therefore, the criterion of subacute rumen acidosis (SARA) was defined as a pH depression below 6.1 for more than 180 min/day rather than pH depression below 5.6 for more than 180 min/day, which was the threshold of previous studies (Gozho, Plaizier, Krause, Kennedy, & Wittenberg, 2005; Plaizier, Krause, Gozho, & Mcbride, 2008). Ruminal pH fluid data were summarized as average pH, time below pH 6.1, and area (time × pH) below pH 6.1 for each 24‐hr period.

### 2.4 Rumen fluid sampling and analysis

The rumen fluid was collected once during each collection period at 4 hr post‐feeding. Rumen fluid was aspirated using an oral probe. After filtering the ruminal fluid with four layers of gauze, it was frozen and preserved at −30°C until the concentration of volatile fatty acid (VFA), ammonium nitrogen (NH_3_‐N), and lipopolysaccharide (LPS) level could be measured.

The VFA composition in the ruminal fluid was analyzed by high‐performance liquid chromatography (CTO‐10AV; Shimazu Corporation, Kyoto, Japan) using the bromothymol blue post‐label method. The concentration of ruminal NH_3_‐N was quantified using an ammonia test (Wako Pure Chemical Industries, Ltd, Tokyo, Japan). The LPS level was measured in accordance with the method described by Hirabayashi et al. (2017).

### 2.5 Milk sampling and analysis

Cows were milked twice daily at 08:30 hr and 16:00 hr, and the quantity of milk was measured daily using a milk meter installed in the milking parlor. Milk samples were collected from six consecutive milkings in each collection period. The samples were stored at 4°C until the fat, protein, lactose, and solid‐non‐fat content in the milk were analyzed by the CombiFOSS milk analyzer (Foss Electric, Hillerød, Denmark).

### 2.6 Statistical analysis

Chemical composition, intake, digestibility, ruminal pH, rumen fermentation characteristics (total concentration of VFA, molar proportions of acetate, propionate, and butyrate, lactic acid concentration, ammonia concentration, and LPS), and lactation performance were analyzed using model equation following the General Linear Model procedure of SAS (JMP^®^ 11; SAS Institute Inc., Cary, NC, USA):

$$Y_{\mathrm{ij}}=\mu +D_{i}+C_{j}+e_{\mathrm{ij}},$$

where *Y*_*ij*_ = dependent variables; μ = overall mean; *D*_*i*_ = effect of the dietary treatment *i*;* C*_*j*_ = effect of the animal *j*; and *e*_*ij*_ = error.

In this model, periodic effects were ignored based on a preliminary analysis.

Rumen fluid pH was analyzed using a mixed procedure in SAS using the following model:

$$Y_{\mathrm{ijk}}=\mu +D_{i}+C_{j}+T_{k}+{\left(D\times T\right)}_{\mathrm{ik}}+e_{\mathrm{ijk}},$$

where *Y*_*ijk*_ = dependent variables; μ = overall mean; *D*_*i*_ = effect of dietary treatment *i*;* C*_*j*_ = effect of the animal *j*;* e*_*ij*_ = main plot error; *T*_*k*_ = effect of time *k* in hours since the diet was offered; (*D* × *T*)_*ik*_ = interaction between diet *i* and time *k*; and *e*_*ijk*_ = subplot error.

## 3 RESULTS AND DISCUSSION

DM, EE, non‐fiber carbohydrates (NFC), and starch concentration were lower (*p *<* *0.01, *p *<* *0.05) in the KP group compared to that in the control group (Table 2). aNDFom concentration was higher (*p *<* *0.01) in the KP group compared to that in the control group. KP contained more aNDFom and less starch than rolled corn. These differences resulted in higher concentration of aNDFom and lower concentration of NFC and starch in the KP group than in the control group. DM, organic matter (OM), CP, EE, aNDFom, and starch digestibility were not significantly affected by the diets. NFC digestibility was higher (*p *<* *0.01) in KP group than in the control group. Consequently, there was no difference in TDN concentration, and TMR included in KP had the same level of digestibility as that of TMR that included the rolled corn. DM and CP intake were not significantly affected by the diets (Table 3). The aNDFom intake was higher (*p *<* *0.05) in the KP group compared to that in the control group. NFC and starch intake were lower (*p *<* *0.05) in the KP group compared to that in the control group. DM, OM, and TDN intake were not significantly affected by the diets. Thus, the amount of TDN supplied was comparable.

**Table 2:** Chemical composition and apparent digestibility of the diet fed to lactating dairy cows in control group and KP group

	Control group	KP group	*SEM*	*p*‐Value
Chemical composition (% DM)				
DM (%)	68.5	63.2	1.1	0.006
Organic matter	94.5	94.4	0.1	0.704
Crude protein	15.3	15.6	0.3	0.773
Ether extract	2.5	2.1	0.1	0.003
aNDFom	39.6	47.0	1.6	0.007
NFC	37.1	29.8	1.6	0.011
Starch	28.7	19.7	1.8	0.012
Digestibility (%)				
Dry matter	63.1	65.7	2.6	0.713
Organic matter	65.9	68.4	2.4	0.693
Crude protein	59.2	60.9	2.9	0.845
Ether extract	48.8	52.1	3.4	0.756
aNDFom	52.2	59.2	3.3	0.502
NFC	83.3	87.2	2.6	0.019
Starch	95.8	97.7	0.7	0.123
Total digestible nutrients (% DM)^a^	63.8	65.9	2.4	0.731

*Notes*.

*SEM*: standard error of the mean; KP: kraft pulp; DM: dry matter; aNDFom: α‐amylase‐treated ash‐free neutral detergent fiber; NFC: non‐fiber carbohydrates.

^a^Digestible organic matter + 1.25 × digestible ether extract.

**Table 3:** Nutrient intake of lactating dairy cows in control group and KP group

	Control group	KP group	*SEM*	*p*‐Value
Nutrient intake (kg/day)				
Dry matter	20.7	20.2	0.39	0.637
Crude protein	3.2	3.2	0.11	0.912
Ether extracts	0.5	0.4	0.03	0.010
aNDFom	7.9	9.4	0.31	0.019
NFC	7.9	6.1	1.25	0.044
Starch	6.3	4.1	0.44	0.013
Total digestible nutrients	13.2	13.2	0.61	0.932

*Notes*.

KP: kraft pulp; *SEM*: standard error of the mean; aNDFom: α‐amylase‐treated ash‐free neutral detergent fiber; NFC: non‐fiber carbohydrates.

**Figure 1:**
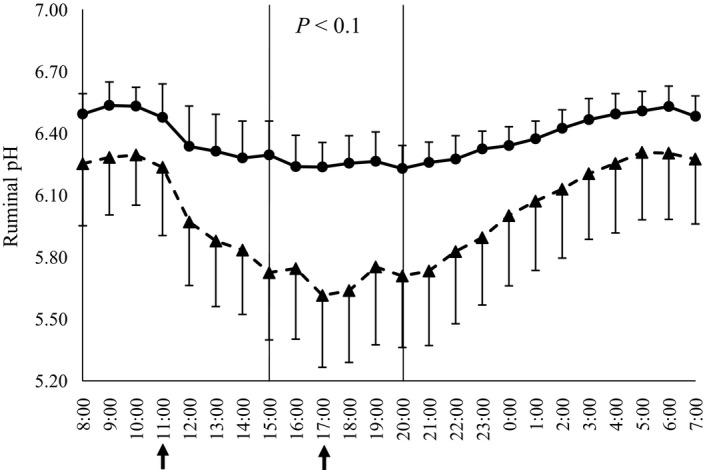
Daily change in ruminal pH of lactating dairy cows in control group (▲) and KP group (●). Arrow indicates time of feeding. Overall treatment effect, *p *= 0.170; time effect, *p *< 0.001; treatment × time interaction, *p *< 0.001. Difference between groups is indicated by *p*‐values. Error bars *SEM*

Both diets showed the same pattern of pH fluctuation; pH decreased after feeding diets and it gradually recovered from the evening to the following morning (Figure 1). Ruminal fluid pH remained higher in the KP group than in the control group. The interaction between diet and time was significant (*p *<* *0.001). The KP group tended to have a higher ruminal fluid pH from 4 to 10 hr after feeding (*p *<* *0.10). The average rumen fluid pH did not differ among the diets (Table 4). However, the duration of a pH below 6.1 and area pH with below 6.1 tended to decrease in the KP group compared to the control group (*p *<* *0.10). The length of time that the KP group had a ruminal fluid pH below 6.1 slightly exceeded the SARA determination criterion. However, the duration of a ruminal fluid pH below 6.1 in the control group was approximately triple that of the SARA determination criterion. It is known that when grains and starch are increased in diets, ruminal fluid pH is reduced and the length of time at a pH below 6.0, 5.8, and 5.6 is increased (Danschar et al., 2015; Gozho et al., 2005; Krause & Oetzel, 2005; Li, Gozho, et al., 2012; Li, Khafipour, et al., 2012). The speed of in vitro rumen fermentation of KP was shown to be mid way between that of rolled corn and hay (Hada et al., 2016). These findings indicate that replacing part of the rolled corn with KP could increase the ruminal fluid pH and suppress the occurrence of SARA.

**Table 4:** Ruminal pH variables of lactating dairy cows in control group and KP group

	Control group	KP group	*SEM*	*p*‐Value
Average pH	5.99	6.39	0.17	0.176
Time < pH 6.1, min/day	576.1	197.5	170.4	0.067
Area < pH 6.1, min × pH/day	56.3	20.0	183.9	0.081

*Notes*.

KP: kraft pulp; *SEM*: standard error of the mean.

Diet had no effect on the concentrations of total VFA, lactic acid in ruminal fluid, or ruminal NH_3_‐N (Table 5). The acetic acid ratio in ruminal fluid was significantly higher in the KP group compared to that of the control group (*p *<* *0.05). The propionate acid ratio in ruminal fluid was significantly lower in the KP group compared to that of the control group (*p *<* *0.05). Diets had no effect on other VFA ratios. As a result, the acetate:propionate (A/P) ratio was increased in the KP group compared to that of the control group (*p *<* *0.05). It was reported that the total VFA concentration of ruminal fluid was not affected by the use of a wood‐based feed that had been subjected to a steaming treatment as well as feeds that had not been treated (Kajikawa et al., 1987; Nakagawa et al., 2010; Sharma, Forsberg, & Guenter, 1979). These studies had replaced part of the roughage with wood‐based feed, but there has been no report mentioning the replacement of part of the concentration feed with wood‐based feed. In this study, the total VFA concentration was not affected by replacing a part of corn with KP because the nutritional value of KP was equivalent to the nutritional value of corn. However, no consistent results have been obtained concerning the VFA composition ratio when it comes to the effect of using a wood‐based feed thus far. Using a wood‐based feed that had undergone a steaming treatment did not affect the VFA composition ratio in Holstein steer cattle (Kajikawa et al., 1987). When untreated, wood‐based feed was fed to Japanese black cattle, the acetic acid ratio decreased and the propionic acid ratio increased, and similar conditions were observed when they were given a high concentrate diet (Nakagawa et al., 2010). While it appears that the type of treatment used for a wood‐based substance affects the VFA composition, our findings differed from previous studies. In addition, previous studies had replaced part of the roughage with wood‐based feed (Kajikawa et al., 1987; Nakagawa et al., 2010), but in the present study, it was replaced with rolled corn. When KP was fed to lactating cows as a replacement for rolled corn, the composition ratio of VFA was similar to that of a high roughage diet.

**Table 5:** Ruminal profiles and rumen LPS of lactating dairy cows in control group and KP group

	Control group	KP group	*SEM*	*p*‐Value
Total VFA (mmol/dl)	10.1	10.2	2.2	0.531
VFA composition (mol %)				
Acetic	56.0	61.9	1.2	0.037
Propionic	31.3	26.5	1.0	0.018
Butynic	12.7	11.6	0.5	0.113
Acetic:Propionic	1.8	2.3	0.1	0.014
Lactic acid	1.7	0.2	0.8	0.468
Ammonia N (mg/dl)	4.8	6.2	0.9	0.549
LPS(EU/ml)	26,399.6	17,755.8	4,007.9	0.057

*Notes*.

LPS: lipopolysaccharide; KP: kraft pulp; *SEM*: standard error of the mean; VFA: volatile fatty acid.

The level of LPS activity in ruminal fluid tended to decrease in the KP group compared to that of the control group (*p *=* *0.06) (Table 5). LPS, one of the endotoxins, is a component of the cell wall of Gram‐negative bacteria that is released into the rumen when these bacteria die (Plaizier, Khafipour, Li, Gozho, & Krause, 2012). A high LPS concentration in ruminal fluid has been shown when the diet contained a large amount of grains or starch (Li, Gozho, et al., 2012; Li, Khafipour, et al., 2012) or when SARA was experimentally induced by increasing the supply of a concentrated diet (Gozho, Karus, & Plaizier, 2006; Gozho et al., 2005; Li, Gozho, et al., 2012; Li, Khafipour, et al., 2012). Furthermore, LPS level increased when the ruminal fluid pH decreased (Gozho et al., 2005). The KP group in the present study had a lower starch content compared to the control group, and as the NDF content was higher, the decline in ruminal fluid pH was suppressed. These factors were thought to have influenced the decrease in LPS level in the ruminal fluid of the KP group.

The milk protein content, milk lactose content, and solid‐non‐fat content were not affected by the diets (Table 6). However, the milk fat content tended to increase in the KP group compared with that in the control group (*p *=* *0.07). When the feed grain ratio was low, the A/P ratio in that ruminal fluid increased and the milk yield decreased (Agle et al., 2010), but the milk fat content increased (Bauman & Griinari, 2003). In the present study, the A/P ratio was increased in the KP group, but no differences were observed in milk yield. Furthermore, the milk fat content of the KP group was higher than that of the control group, but the difference was not statistically significant. Generally, when the NDF content in TMR is increased, TDN content tends to decrease. However, KP did not decrease the TDN content of TMR in spite of a higher NDF content. Therefore, this was speculated to be the reason why the milk yield of the KP group was similar to the control group in this study.

**Table 6:** Milk production of lactating dairy cows in control group and KP group

	Control group	KP group	*SEM*	*p*‐Value
Milk yield (kg/day)	24.9	23.4	1.4	0.521
Milk composition (%)				
Fat	4.38	5.05	0.23	0.069
Protein	3.94	3.85	0.08	0.444
Lactose	4.56	4.47	0.05	0.176
Solid‐non‐fat	9.51	9.37	0.08	0.230

*Notes*.

KP: kraft pulp; *SEM*: standard error of the mean.

In conclusion, replacing 50% of rolled corn DM with KP did not affect DM intake, digestibility, or milk yield of lactating dairy cows. Feeding KP not only increased the ruminal fluid pH, but also reduced the occurrence of SARA and contributed to the depression of ruminal fluid LPS level. In addition, feeding KP had the effect of increasing the milk fat content. Therefore, we conclude that KP could be a valuable replacement for rolled corn in feed, and replacing 50% of rolled corn with KP could improve the rumen fermentation in lactating dairy cows without lowering milk productivity.
